# Estimating contact rates at a mass gathering by using video analysis: a proof-of-concept project

**DOI:** 10.1186/1471-2458-14-1101

**Published:** 2014-10-24

**Authors:** Jeanette J Rainey, Anil Cheriyadat, Richard J Radke, Julie Suzuki Crumly, Daniel B Koch

**Affiliations:** Division of Global Migration and Quarantine, Centers for Disease Control and Prevention, Atlanta, GA USA; Oak Ridge National Laboratory, Oak Ridge, TN USA; Department of Electrical, Computer, and Systems Engineering, Rensselaer Polytechnic Institute, Troy, NY USA; Oak Ridge Associated Universities, Oak Ridge, TN USA

**Keywords:** Mass gathering, Video analysis, Contact rates, Social mixing, Simulation

## Abstract

**Background:**

Current approaches for estimating social mixing patterns and infectious disease transmission at mass gatherings have been limited by various constraints, including low participation rates for volunteer-based research projects and challenges in quantifying spatially and temporally accurate person-to-person interactions. We developed a proof-of-concept project to assess the use of automated video analysis for estimating contact rates of attendees of the GameFest 2013 event at Rensselaer Polytechnic Institute (RPI) in Troy, New York.

**Methods:**

Video tracking and analysis algorithms were used to estimate the number and duration of contacts for 5 attendees during a 3-minute clip from the RPI video. Attendees were considered to have a contact event if the distance between them and another person was ≤1 meter. Contact duration was estimated in seconds. We also simulated 50 attendees assuming random mixing using a geo-spatially accurate representation of the same GameFest location.

**Results:**

The 5 attendees had an overall median of 2 contact events during the 3-minute video clip (range: 0–6). Contact events varied from less than 5 seconds to the full duration of the 3-minute clip. The random mixing simulation was visualized and presented as a contrasting example.

**Conclusion:**

We were able to estimate the number and duration of contacts for 5 GameFest attendees from a 3-minute video clip that can be compared to a random mixing simulation model at the same location. The next phase will involve scaling the system for simultaneous analysis of mixing patterns from hours-long videos and comparing our results with other approaches for collecting contact data from mass gathering attendees.

## Background

During the response to the emerging 2009 H1N1 influenza pandemic, public health officials leveraged infectious disease models to develop a range of pandemic scenarios to explore the impact of social distancing measures on mitigating influenza transmission. However, essential components of infectious disease models were frequently based on constant or random contact rates and mixing patterns of infectious and susceptible members of the population. Better quantification of individual contact rates and mixing patterns will become increasingly important as public health officials require more accurate infectious disease models for refining or validating pandemic mitigation strategies, including decisions regarding when to postpone or cancel mass gatherings [1].

Mass gatherings can create environments highly conducive for influenza transmission through virus-containing respiratory droplets and possibly through fomites, due to the spatial and temporal congregation of infectious and susceptible individuals [2]. Factors that commonly determine the effectiveness of transmission include the level of virus circulation, population susceptibility, and the intensity and duration of social mixing at the mass gathering [3, 4]. As mixing patterns at conventions, sporting events, and festivals are unlikely to be homogenous, a better understanding of this variability would greatly facilitate decision-making regarding the public health risk of mass gatherings during a pandemic. A number of approaches have been used to estimate mixing patterns at mass gatherings with variable levels of success, primarily due to the need for high participation among attendees and challenges associated with recording face-to-face interactions in a spatially and temporally accurate way [5–8].

New techniques for analyzing video recordings have recently been used for evaluating vehicular traffic flow and airport security [9–14]. The success of these projects suggests that video analysis could also be used to address gaps in quantifying mixing patterns at mass gatherings. In this article, we describe our proof-of-concept project on the use of video analysis techniques to estimate contact rates of attendees of the 2013 GameFest event at Rensselaer Polytechnic Institute (RPI) in Troy, New York. We also present a geo-spatially accurate random mixing simulation of the same GameFest location.

## Methods

### Data collection

We used arrays of networked video cameras to record attendees of the GameFest event at RPI in Troy, NY. The event, which was held in RPI’s Experimental Media and Performing Arts Center (EMPAC) on April 26-27, 2013, had approximately 400 attendees. GameFest provided the chance for RPI students to demonstrate their work in game design and simulations to the public, the gaming industry, and other students and faculty.

EMPAC is a multipurpose facility with several large studio rooms and auditoriums used for concerts, art installations, research projects, and public events. Eight ceiling-mounted video cameras were placed at two locations (four in Studio 1 and four in the mezzanine) where participants visited display booths according to their level of interest. The video cameras were calibrated to capture distinct but overlapping fields of view such that the entire space in each location was recorded. Each camera recorded 4 hours of video on April 27, the second day of the event.

### Analysis

For this proof-of-concept project, we randomly selected a 3-minute clip of video recordings from Studio 1 for analysis. Video analysis techniques were used to detect and track multiple subjects by measuring the optical flow of low-level features (corners, edges, lines, color, and texture) using Matlab programming [9–13]. The tracking approach identifies group structures formed in the crowd, updates the structure configurations continuously, and tracks subjects in a unique way that preserves the structural configuration. Each subject’s motion path and interaction data (contact frequency and statistics on spatial proximity) are then extracted. We provide more detail in the supplement to this article and in Yan *et al.* [13]. Contact data were computed for 5 GameFest attendees. An attendee was considered to have a contact event if the distance between the attendee and another person was ≤1 meter.

In this proof-of-concept project, we also explored the use of IMPACT, an existing Oak Ridge National Laboratory (ORNL) platform, for conducting random mixing simulations [15], including the use of the application’s drawing tools to outline the boundaries of Studio 1 in a geospatially-accurate way. In this simulation, we introduced 50 participants to Studio 1 using a uniform X:Y distribution for their starting point, with proximity between participants determined by each participant’s random direction (0 to 360 degrees) and step size based on a Gaussian distribution at one-second intervals. Physical barriers in Studio 1 including the display booths restricted participants’ movements and contact with other participants.

As with other public venues at RPI, EMPAC uses video cameras for security purposes. Therefore, the attendees at GameFest had no assumptions of privacy. The project video cameras were ceiling-mounted, and the low-level feature tracking could not be used to identify attendees. Since the attendees were only imaged from above at a public event, and were not personally identifiable, no consent was collected from the attendees, and their motions were natural. We received permission for camera installation and filming from EMPAC staff and GameFest event organizers. This project was reviewed and approved by the Institutional Review Boards at RPI and Oak Ridge Associated Universities. The Human Subjects Research Office at the Centers for Disease Control and Prevention determined that this was an evaluation project and, therefore, exempt from IRB review.

## Results

**Figure 1:**
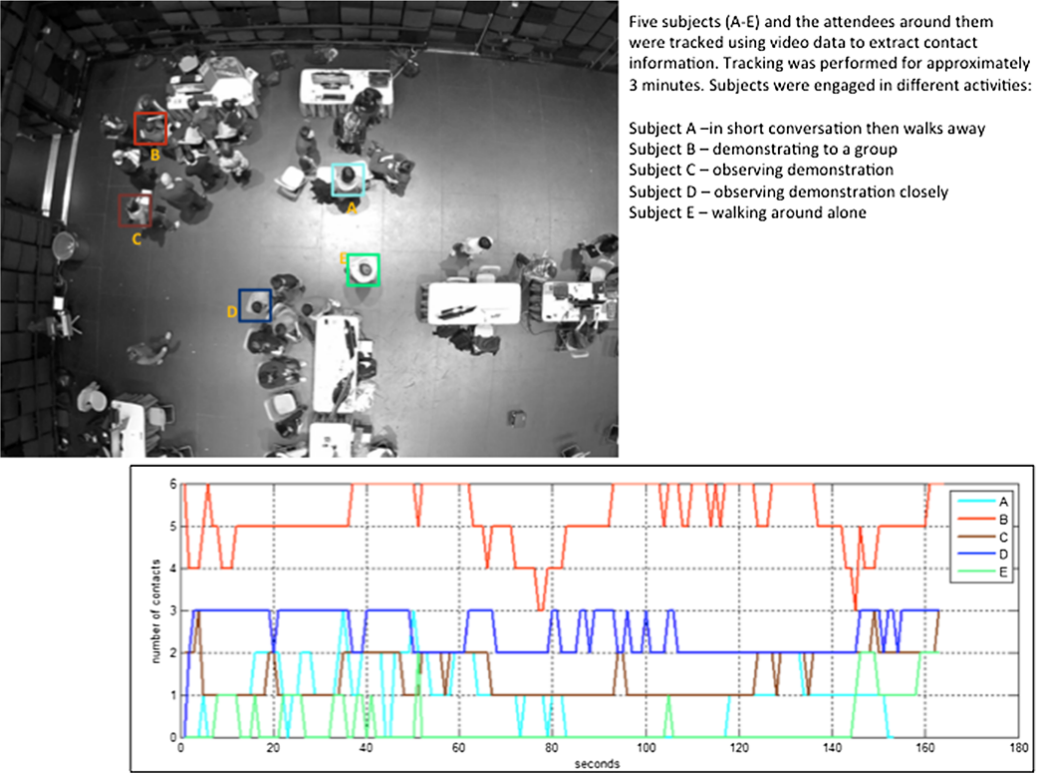
**Attendees captured during three-minute video clip, GameFest event, April 2013, with the number and duration of contacts of 5 attendees (A-E) during the clip.** A contact event was defined as contact between attendees and any other person within a distance ≤1meter.

**Figure 2:**
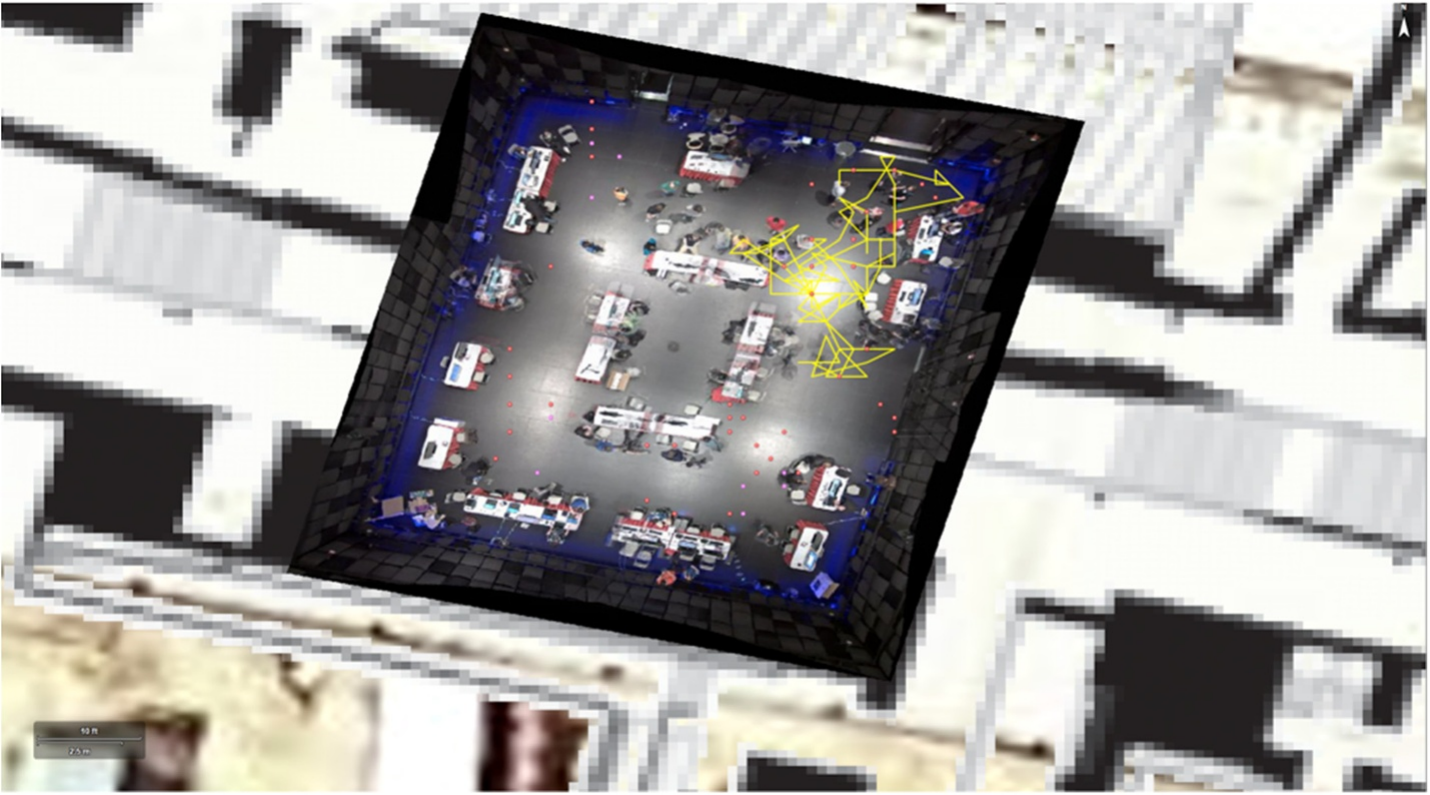
**Snapshot of simulation of 2013 GameFest event (Studio 1), Rensselaer Polytechnic Institute, Troy, NY, April 2013, using IMPACT computer application.** Yellow tracks reflect random mixing of a sample of participants at one-second intervals during a three-minute video clip. Participants were introduced using a uniform X:Y distribution from their starting point, with proximity between participants determined by each participant’s random direction (0 to 360 degrees) and step size based on a Gaussian distribution. Physical barriers in Studio 1 restricted participants’ movements and contact with other participants.

The 5 attendees had an overall median of 2 contact events during the 3-minute video clip (range: 0-6) (Figure 1). Contact events were typically not continuous, varying from less than 5 seconds to the full duration of the 3-minute clip. The number of contact events differed among the five attendees, but were relatively consistent for each attendee across the duration of the clip (Table 1). Figure 2 shows a snapshot from the end of a 3-minute simulation of 50 attendees in a geo-spatially accurate representation of Studio 1, assuming random mixing among GameFest attendees.

**Table 1:** **Median number of contacts per minute for 5 attendees during a 3-minute video clip at the GameFest event, April 2013**

Minutes in video	Median number of contacts				
	A - Light blue	B - Red	C - Brown	D - Dark blue	E - Green
**1**	1	5	1	3	0
**2**	0	5	1	2	0
3*	1	5	2	2	0

*Minute three includes 43 seconds (total video clip = 163 seconds).A contact event was defined as a contact between the attendee and any other person within a distance ≤1meter (subjects are denoted by color codes as shown in Figure 1).

## Discussion

Due to existing limitations in capturing mixing patterns at mass gatherings, we developed a proof-of-concept project to assess the use of video recordings for estimating contact rates at the GameFest event in Troy, New York, in 2013. We used video analysis to estimate the number and duration of contacts for 5 event attendees. The project suggests that video tracking and analysis may be feasible for generating contact parameters in a spatially and temporally accurate way, but more work is needed to assess this approach in larger and more diverse mass gathering settings [8, 16, 17].

Most noteworthy is that we were able to record 100% of attendees within each selected location at the GameFest event, while avoiding direct contact with or creating additional burden for the attendees. This is a possible advantage compared to other approaches, including the use of remote sensors, for to capturing contact data from mass gathering attendees. Remote sensors are small devices, worn on a lanyard or belt that can capture interactions with others also wearing the device, and have been used to generate social mixing patterns in school settings. However, a large proportion of the target population is typically needed in order to accurately describe person-to-person interactions, which is dependent on volunteer participation. Researchers using radiofrequency identification devices to capture contact data from conference participants in France achieved a 30% participation rate [5]. Due to the current lack of empirical data, researchers are unable to assess if volunteers and non-volunteers (who may prefer not to be tracked) have different social mixing behaviors [8].

Remote sensors and similar devices can also get lost, damaged, or lose power, all potentially impacting data quality [8]. Though video cameras can get damaged or lose data, this and related constraints are likely to be rare events. Additionally, our approach could be applied to existing video recordings obtained from large mass gatherings for other purposes, such as public security, further minimizing data collection challenges; however, the use of existing video recordings could require additional calibration and processing post-collection. Since video recordings are commonly used for security purposes at most public venues, there will be fewer concerns with privacy.

For the proof-of-concept project, we defined a contact event if the distance between the selected attendee and another person was ≤1 meter. Using this definition, our 5 attendees had a median of 2 contact events during the 3-minute video clip. Coding changes in Matlab programming for further analysis of the video recordings can increase the contact definition to less than or equal to 2 meters, the commonly used threshold for influenza transmission through respiratory droplets [2]. Video analysis allows the user to modify the contact definition after data collection but also to explore qualitative mixing behavior commonly not collected through current contact data collection tools. In low-density mass gatherings, information on physical contact (e.g., a handshake or kiss) as well as closeness of contact could be quantified and eventually added to simulation models.

Age is an important predictor for a number of infectious diseases, including influenza [18]. During the recent Influenza A(H1N1) pandemic in 2009, the majority of reported cases in the United States occurred in the school-aged population [19, 20]. Older-aged cohorts experienced lower attack rates, presumably due to residual immunity from previous infections with genetically similar influenza viruses [21, 22]. Our approach using video analysis was not able to generate age-specific contact data that would be required for influenza transmission models. Contact surveys (either web-based or paper) and the distribution of remote sensing devices allow researchers to collect demographic information on participants [6, 7]. To address the limitation, general information on attendees (e.g., age, place of residence, and duration of stay) could be obtained from mass gathering organizers and proportionally distributed across attendees identified through video analysis.

While video analysis could provide important information on social mixing at mass gatherings, computer capacity could be a constraining factor [14, 23]. Our video analysis relied on a multi-person tracking algorithm based on hierarchical group structures to track subjects along with the other participants in the scene. Video analysis for this proof of concept project was performed on a single desktop computer. Automated video analysis for the entire video data (~40 hours of video) could require parallel implementations of the algorithm or executing the algorithm on a high-performance computing platform. High-density mass gatherings such as the Hajj or the Olympics could require extremely high-resolution cameras and more complex feature tracking, increasing the computational demand. Analysis of existing video recordings from such events could assist in evaluating the potential error rate (e.g., losing unique objects or merge and split events) in tracking individuals in such densely populated environments [9–14].

Our proof-of-concept project used a 3-minute clip from a single video camera. The ability to stitch together the frames and track participants across multiple cameras with a high level of precision will be required [9–14]. This work is currently in progress for the eight cameras used at the Game Fest event. Following this process, we will be able to simultaneously track attendees and estimate the number and duration of contacts at different time frames across the two event locations (Studio 1 and the Mezzanine). A previously implemented real-time airport security checkpoint surveillance system using a camera network demonstrates the feasibility of this approach [14]. In this surveillance system, a network of 19 cameras was used to track airline passengers and their carry-on bags through security. The system was robust to populated and complex interactions common to mass transit settings [14].

Scaling up our video analysis will focus on identifying key locations and time frames likely to be representative of social mixing patterns across the mass gathering venue. Complete and simultaneous coverage (of all attendees for the full duration of the gathering) is likely infeasible due to computational requirements. However, for the purpose of estimating contact rates for modeling infectious disease transmission, complete attendee coverage may not be required. The durations of contacts across a subset of mass gathering attendees (rather than the full social network) could be sufficient to explain infectious disease transmission dynamics [5, 16].

Following completion of the analysis of the GameFest event video recordings and refinement of the automated analysis process, we propose to implement a larger study to compare the video analysis results with other approaches for contact rate estimation. Assessment of mixing patterns in school settings has used a combination of contact surveys and remote sensors over one or more days [6, 24]. We propose to use a similar combination of video recordings and remote sensors along with the collection of demographic information to compare and contrast contact characteristics of mass gathering attendees. To ensure the appropriate linkage between the two sources of contact data for individual attendees, we will select a small subset of volunteer attendees to wear a specifically designed marker (e.g., hat or jacket) that will facilitate identification of this individual in the video recordings. A small experiment at EMPAC or another location will be implemented in advance to test the linkage process.

The results of the initial IMPACT simulation of attendee interactions assuming random mixing in Studio 1 can serve as a baseline and be compared with future simulations using statistical distributions of the number and duration of contacts derived from the analysis of video recordings from GameFest. These results have the potential to help public health professionals determine whether precise contact parameter estimates are needed for simulations, or whether assumptions of random mixing could provide valid approximations for exploring infectious disease transmission at certain types of gatherings (e.g., professional conferences versus sporting events). IMPACT allows users to modify both micro- and macro- environments of a real or hypothetical setting, and therefore can be used for simulating mixing patterns and transmission dynamics at other mass gatherings [15–17].

Available evidence suggests that restricting mass gatherings in addition to implementing other social distancing measures (e.g., school closures) could help mitigate pandemic influenza transmission [25]. This evidence is primarily based on surveillance reports and outbreak investigations that rely on case detection with limited differentiation between types of mass gatherings. Projects to better estimate social mixing at mass gatherings, such as our proof-of-concept project, may help provide insight on transmission dynamics and generate information through computer simulations on the probabilities of pandemic propagation at and after the gathering, as well as describe the variability in these outputs by type of mass gathering (i.e., venue, purpose, size, and duration) [16, 17, 25–27]. A combination of approaches will likely be needed to capture the complex social mixing patterns at mass gatherings.

## Conclusion

Our project demonstrated the use of video analysis to estimate contact rates of 5 attendees at a mass gathering. The next phase of work will involve scaling the system for simultaneous analysis of hours-long video recordings. Comparing the results of our approach with other methods for contact rate estimation could assist in further refinement of our video analysis techniques. The generated contact parameters may help improve computer simulations of influenza transmission at a mass gathering using IMPACT or other modeling applications, with the goal of identifying effective prevention and control strategies, including whether a mass gathering should be postponed or cancelled during a pandemic.

## References

[1] CDC (2014). Interim CDC Guidance for Public Gatherings in Response to Human Infections With Novel Influenza A (H1N1), 2009.

[2] Collignon PJ, Carnie JA (2006). Infection control and pandemic influenza. Med J Aust.

[3] Rashid H, Haworth E, Shafi S, Memish ZA, Booy R (2008). Pandemic influenza: mass gatherings and mass infection. Lancet Infect Dis.

[4] Abubakar I, Gautret P, Brunette GW, Blumberg L, Johnson D, Poumerol G, Memish ZA, Barbeschi M, Khan AS (2012). Global perspectives for prevention of infectious diseases associated with mass gatherings. Lancet Infect Dis.

[5] Stehlé J, Voirin N, Barrat A, Cattuto C, Colizza V, Isella L, Régis C, Pinton JF, Khanafer N, Van den Broeck W, Vanhems P (2011). Simulation of an SEIR infectious disease model on the dynamic contact network of conference attendees. BMC Med.

[6] Smieszek T, Barclay VC, Seeni I, Rainey JJ, Gao H, Uzicanin A, Salathé M (2014). How should social mixing be measured: comparing web-based survey and sensor-based methods. BMC Infect Dis.

[7] Read JM, Edmunds WJ, Riley S, Lessler J, Cummings DA (2012). Close encounters of the infectious kind: methods to measure social mixing behaviour. Epidemiol Infect.

[8] Cattuto C, Van den Broeck W, Barrat A, Colizza V, Pinton JF, Vespignani A (2010). Dynamics of Person-to-Person Interactions from Distributed RFID Sensor Networks. PLoS One.

[9] Javed O, Rasheed Z, Shafique K, Shah M (2003). Tracking across multiple cameras with disjoint views. Proc on 9th Int Conference Comput Vision.

[10] Cheriyadat AM, Radke RJ (2008). Detecting dominant motion in dense crowds. J Special Topics Signal Process.

[11] Cheriyadat AM, Bhaduri BL, Radke RJ (2008). Detecting Multiple Moving Objects in Crowded Environments With Coherent Motion Regions. Proc. On IEEE Computer Society Conference on Computer Vision and Pattern Recognition Workshops.

[12] Ali S, Shah M (2008). Floor fields for tracking in high density crowd scenes. Proc 10th Eur Conference Comput Vision.

[13] Yan X, Cheriyadat A, Shah SK (2014). Hierarchical Group Structures in Multi-Person Tracking. Proc. of the 22nd IEEE International Conference on Pattern Recognition, Stockholm, Sweden.

[14] Wu Z, Radke RJ (2011). Real-Time Airport Security Checkpoint Surveillance Using a Camera Network. Workshop on Camera Networks and Wide Area Scene Analysis, in Conjunction With CVPR.

[15] Koch DB, Payne PW (2012). An Incident Management Preparedness and Coordination Toolkit. Proc. of the 2012 IEEE Global Humanitarian Technology Conference, Seattle, Washington.

[16] Chowell G, Nishiura H, Viboud C (2012). Modeling rapidly disseminating infectious disease during mass gatherings. BMC Med.

[17] Johansson A, Batty M, Hayashi K, Al Bar O, Marcozzi D, Memish ZA (2012). Crowd and environmental management during mass gatherings. Lancet Infect Dis.

[18] Monto AS (1999). Interrupting the transmission of respiratory tract infections: theory and practice. Clin Infect Dis.

[19] Kwok KO1, Cowling BJ1, Wei VW1, Wu KM1, Read JM2, Lessler J3, Cummings DA3, Peiris JS4, Riley S5 (2014). Social contacts and the locations in which they occur as risk factors for influenza infection. Proc Biol Sci.

[20] Shrestha SS, Swerdlow DL, Borse RH, Prabhu VS, Finelli L, Atkins CY, Owusu-Edusei K, Bell B, Mead PS, Biggerstaff M, Brammer L, Davidson H, Jernigan D, Jhung MA, Kamimoto LA, Merlin TL, Nowell M, Redd SC, Reed C, Schuchat A, Meltzer MI (2011). Estimating the burden of 2009 pandemic influenza A (H1N1) in the United States (April 2009-April 2010). Clin Infect Dis.

[21] Reed C1, Katz JM, Hancock K, Balish A, Fry AM, H1N1 Serosurvey Working Group (2012). Prevalence of seropositivity to pandemic influenza A/H1N1 virus in the United States following the 2009 pandemic. PLoS One.

[22] Hancock K, Veguilla V, Lu X, Zhong W, Butler EN, Sun H, Lui F, Dong L, DeVos JR, Gargiuollo PM (2009). Cross-reactive antibody responses to 2009 pandemic H1N1 influenza virus. N Engl J Med.

[23] Haritaoglu I, Harwood D, David LS (2000). Real-time surveillance of people and their activities. IEEE Tansac Pattern Analysis Machine Intell.

[24] Barclay VC, Smieszek T, He J, Cao G, Rainey JJ, Gao H, Uzicanin A, Salathé M (2014). Positive network assortativity of influenza vaccination at a high school: implications for outbreak risk and herd immunity. PLoS One.

[25] Ishola DA, Phin N (2011). Could influenza transmission be reduced by restricting mass gatherings? Towards an evidence-based policy framework. J Epidemiol Glob Health.

[26] Shi P, Keskinocak P, Swann JL, Lee BY (2010). The impact of mass gatherings and holiday traveling on the course of an influenza pandemic: a computational model. BMC Public Health.

[27] Khan K, McNabb SJ, Memish ZA, Eckhardt R, Hu W, Kossowsky D, Sears J, Arino J, Johansson A, Barbeschi M, McCloskey B, Henry B, Cetron M, Brownstein JS (2012). Infectious disease surveillance and modeling across geographic frontiers and scientific specialties. Lancet Infect Dis.

[CR28] The pre-publication history for this paper can be accessed here:http://www.biomedcentral.com/1471-2458/14/1101/prepub

